# Case Report: A case series of using whole exome sequencing to detect novel variants in Vietnamese patients with inborn errors of immunity

**DOI:** 10.3389/fgene.2026.1818952

**Published:** 2026-05-28

**Authors:** Nguyen Thi Kim Lien, Nguyen Van Tung, Le Thi Minh Huong, Nguyen Thi Van Anh, Nguyen Thi Phuong Mai, Tran Minh Dien, Nguyen Thanh Hien, Nguyen Thien Tao, Nguyen Huy Hoang

**Affiliations:** 1 Institute of Biology, Vietnam Academy of Science and Technology, Hanoi, Vietnam; 2 Vietnam National Hospital of Pediatrics, Ministry of Health, Hanoi, Vietnam; 3 Allergy, Immunology and Rheumatology Department, Vietnam National Hospital of Pediatrics, Ministry of Health, Hanoi, Vietnam; 4 Human Genetics Department, Vietnam National Hospital of Pediatrics, Ministry of Health, Hanoi, Vietnam; 5 Faculty of Biology, Graduate University of Science and Technology, Vietnam Academy of Science and Technology, Hanoi, Vietnam

**Keywords:** hyper IgE, inborn errors of immunity (IEIs), primary immunodeficiency (PID), severe combined immunodeficiency (SCID), Vietnamese patients, whole exome sequencing (WES), XLA

## Abstract

**Background:**

Inborn errors of immunity (IEIs) are rare diseases that affect the immune system. Variants in over 500 genes have been identified as causative of 555 IEIs, with clinical phenotypes that can be heterogeneous within the same gene or even within the same variant. Therefore, these challenges make it difficult to determine the cause of IEI in individuals with immune disorders and to link clinical phenotypes to the precise genetic damage. An incorrect diagnosis can miss approximately 25% of IEI patients with overlapping initial manifestations. Accurate diagnosis and timely treatment are essential to improving quality of life and prolonging the lives of patients, as these patients often suffer from severe, life-threatening infections if left untreated.

**Methods:**

In this study, whole exome sequencing (WES) was used to identify potentially pathogenic variants in six Vietnamese IEI patients. Variants in genes associated with IEIs were screened from WES data using criteria for known and/or novel pathogenic variants, and variants with minor allele frequency (MAF) < 0.001. The pathogenicity of the novel variant was assessed using The American College of Medical Genetics and Genomics (ACMG) criteria and *in silico* predictive software.

**Results:**

Six variants were identified as causative in six study patients, including c.116–2A>G (in the *IL2RG* gene); c.215delA, p.Asn72Ilefs*49 and c.83G>A, p.Arg28His (in the *BTK* gene); c.1110–3C>A (in the *STAT3* gene); c.1114G>A, p.Glu372Lys (in the *STAT6* gene); and c.526C>T, p.Arg176* (in the *NHEJ1* gene). Of these, the variant c.1110–3C>A in the *STAT3* gene was newly identified in an IEI patient.

**Conclusion:**

Although this study has limitations in analyzing the WES of six patients with different types of IEI, the results will contribute to a better understanding of the genetic causes of IEIs. Furthermore, the study emphasizes the importance of accurate diagnosis, which helped improve treatment outcomes and enhance the quality of care for individuals with IEIs.

## Introduction

Inborn errors of immunity (IEIs), also known as primary immunodeficiency diseases (PID), are a group of rare genetic diseases caused by defects in the immune system. IEIs represent a growing group of diseases characterized by a diverse combination of severe, recurrent infections. IEI patients with complex clinical phenotypes require careful analysis to diagnose the cause of the disease. Early diagnosis of IEIs can lead to effective interventions for life-saving or life-changing; however, this remains a challenge due to the rarity of the disease, which can lead to delayed diagnosis and a worse prognosis (Ponsford et al., 2018).

IEIs are primarily a group of monogenic disorders caused by genes involved in immune defense and immune regulation. Affected individuals typically exhibit increased susceptibility to infections, recurrent infections, autoimmunity, inflammatory manifestations, and allergic symptoms (Castagnoli et al., 2021; Kostova et al., 2023). The pathogenesis of IEI is complex and is strongly dependent on the nature of the variant, as well as the genes and inheritance pattern (Poli et al., 2025). Accordingly, single-allele variants can cause disease due to deficiency of a single allele, negative dominance, or gain of function (GOF). Conversely, biallelic genetic lesions (homozygous, combined heterozygous) cause autosomal recessive (AR) traits due to loss of expression, loss of function (LOF), GOF, or even new functions of proteins encoded by these genes, and X-linked recessive traits arise from LOF or GOF variants on the X chromosome, in hemizygous in males or homozygous in females (Bousfiha et al., 2018; Bousfiha et al., 2020; Picard et al., 2018; Tangye et al., 2020).

The International Union of Immunological Societies (IUIS) currently classifies IEI into 10 main groups, with forms having overlapping phenotypes, including: (1) Severe combined immunodeficiencies (SCID) with 10 genes associated with T–B + SCID, 10 genes for T–B– SCID, and 51 genes for CID; (2) Combined immunodeficiencies with syndromic features with 62 genes and 15 genes with hyper IgE; (3) Predominantly antibody deficiencies with 44 genes; (4) Diseases of immune dysregulation with 71 genes; (5) Congenital defects of phagocytes with 46 genes; (6) Defects in intrinsic and innate immunity with 84 genes; (7) Autoinflammatory diseases with 63 genes; (8) Complement deficiencies with 32 genes; (9) Bone marrow failure with 43 genes; and (10) Phenocopies of inborn errors of immunity with 13 genes (Tangye et al., 2020; Poli et al., 2025; Mahmood, 2025). IEIs are phenotypically complex due to the involvement of multiple genes, and the discovery of many new genes continues to pose challenges for diagnosis and treatment (Yaakoubi et al., 2023). Over the past decade, molecular diagnostics based on next-generation sequencing (NGS) have made a large contribution to the diagnosis of IEIs (Lee and Abraham, 2021).

### Severe combined immunodeficiencies (SCID)

In this group, X-linked severe combined immunodeficiency (X-SCID, OMIM#312863) is a life-threatening IEI that accounts for half of all SCID cases (Belaid et al., 2021). X-SCID is caused by variants in the *IL2RG* gene (OMIM#308380) that is located on the X chromosome at position q13.1 and encodes the interleukin common gamma chain (IL-2Rg or gc) of cytokine receptors for interleukin IL-2, IL-4, IL-7, IL-9, IL-15, and IL-21. Most infants with SCID die within the first year of life if not treated with hematopoietic stem cell transplantation (HSCT), due to severe and recurrent infections as early as the first few months, accompanied by diarrhea and growth retardation. Infections can be caused by viruses, bacteria, and/or fungi, and the BCG vaccine may lead to the spread. The *IL2RG* gene comprises eight exons that encode the common gamma chain (IL-2Rg or gc, also known as CD132), which is part of the high-affinity IL-2 receptor and several interleukin receptors, including IL-4, IL-7, IL-9, IL-15, and IL-21 (Yrina et al., 2008). IL-2Rg plays a crucial role in lymphocyte proliferation and differentiation; therefore, variants in the *IL2RG* gene that result in the absence of T cells, natural killer (NK) cells, and B cells with the T−B + NK− phenotype are associated with dysfunction in the γC-JAK3-STAT5 signaling pathway (Weinstein et al., 2016). To date, more than 200 variants in the *IL2RG* gene have been identified; the majority are single-base changes (missense and nonsense variants), followed by splice site variants, deletions, and insertions (Lim et al., 2019). More recently, variants of the *IL2RG* gene that impair function have been found as the cause of atypical SCID with a milder phenotype (Hou et al., 2022).

Cernunnos defect (OMIM#611291), or Cernunnos-XLF deficiency, is a rare form of SCID caused by variants in the *NHEJ1* gene, leading to impaired DNA repair. The *NHEJ1* gene (OMIM#611290), located on chromosome 2q35, is involved in the nonhomologous end-joining (NHEJ) pathway of DNA repair (Slatter and Gennery, 2020). Variants in the *NHEJ1* gene have been shown to lead to severe immunosuppression (due to decreased T- and B-cells), growth retardation, recurrent infections, and cellular sensitivity to ionizing radiation, similar to that in patients with Nijmegen breakage syndrome (NBS) and DNA ligase IV (LIGIV) syndrome (Buck et al., 2006). In the latest IUIS classification, Cernunnos defect is classified as T−B−NK + SCID (OMIM#611291) (Bousfiha et al., 2020). To date, 16 variants have been updated in the Human Gene Mutation Database (HGMD).

### Hyper IgE

Hyper IgE syndrome (HIES, OMIM#147060) is classified as a subgroup of IEIs, manifesting with a range of symptoms including recurrent infections accompanied by elevated serum IgE levels. Clinical manifestations include recurrent eczema, frequent bacterial infections, mucosal candidiasis, skin rashes that often appear within the first week of life, recurrent pneumonia, cold abscesses caused by *Staphylococcus aureus*, and non-healing skin lesions. The incidence of hyper IgE is approximately 1 in 100,000 live births, with an equal rate between males and females (Joshi et al., 2009; Mogensen, 2013; Hashemi et al., 2017). The most studied form, STAT3-HIES (OMIM#147060), also known as Job syndrome, is a type of HIES with an incidence of less than 1 in 1,000,000 live bỉrths. To date, only 250 cases have been reported worldwide. STAT3-HIES is associated with variants in the signal transducer and activator 3 (*STAT3*) gene (OMIM#102582), located on chromosome 17 (Beziat et al., 2018; Tsilifis et al., 2021), with 180 variants reported in HGMD.

Other forms of HIES associated with different genes have been reported, including HIES2 (OMIM#243700), related to the *DOCK8* gene; HIES3 (OMIM#618282), associated with the *ZNF341* gene; HIES4A (OMIM#619752) and HIES4B (OMIM#618523), both derived from the *IL6ST* gene; and HIES5 (OMIM#618944), caused by the *IL6R* gene (Tsilifis et al., 2021). In addition, other forms of IEIs have been reported to be associated with abnormally high IgE levels. These include SCID and other defects in T cell development, and early identification of the cause can save lives and help elucidate the genetic defects of extreme IgE forms of the disease (Ben-Ali et al., 2019; Tavakol et al., 2019). Single-gene defects leading to severe allergic disease, known as primary allergic disorders (PADs), are among the forms of IEIs reported to be associated with extreme elevation of IgE; however, other forms of IEI with extreme IgE elevation have mostly unknown etiology (Lyons and Milner, 2018; Vaseghi-Shanjani et al., 2021). Currently, only a few IEIs are known to cause severe allergic disease (Milner, 2020). STAT6 (transcriptional signal transducer and activator 6), encoded by the *STAT6* gene (OMIM#601512), is a transcription factor that plays a central role in the pathophysiology of HIES6 (OMIM#620532). STAT6 is a key transcription factor that regulates the biological functions of IL-4, an essential cytokine for type 2 T cell differentiation, survival, proliferation, and IgE class switching in B cells (Goenka and Kaplan, 2011; Villarino et al., 2017; 2020), as well as IL-13, a cytokine involved in anaphylactic shock (Gowthaman et al., 2019). Affected individuals exhibit severe, early-onset, sometimes fatal, multisystem allergic disease that is resistant to conventional treatments. However, therapies targeting the STAT6 signaling pathway have shown remarkable therapeutic efficacy in patients. To date, five variants of the *STAT6* gene have been reported to the HGMD.

### X-linked agammaglobulinemia (XLA)

X-linked immunoglobulin deficiency (XLA, OMIM#300755), a form of the predominantly antibody deficiency disorders, is characterized by recurrent infections due to antibody deficiency resulting from disruption of B lymphocyte development (Conley et al., 2009). Reports from various countries and ethnic groups indicate that approximately 90% of men with XLA carry variants in the *BTK* gene (Chen et al., 2016). The *BTK* gene (OMIM#300300), encompassing 37.5 kb including 19 exons and located on the X-chromosome (Xq21.3 - Xq22), was identified to be related to XLA. The *BTK* gene encodes the B-cell protein tyrosine kinase (Bruton tyrosine kinase), which comprises of five distinct structural domains; Pleckstrin homology (PH), Techomology (TH), Src homology (SH3), SH2, and catalytic kinase (SH1) domains. Both *in vivo* and *in vitro* studies demonstrate that the BTK protein is essential for the survival, cell cycle progression, and proliferation of B cells upon stimulation of surface antigen receptors. Defective BTK impairs early B cell development, leading to a significant reduction in mature B cells in peripheral blood. The crucial role of BTK in B cell development is demonstrated by the widespread B cell deficiency (<2%) and the absence of B progenitor cell differentiation in the bone marrow in patients with pathogenic variants. Lymphocytes in the blood and tissues fail to produce plasma cells, and the production of all immunoglobulins is severely reduced, resulting in a markedly defective antibody response (Aadam et al., 2016; Alvarez-Marquez et al., 2018). Patients with XLA have high morbidity and mortality rates despite advances in diagnosis and treatment (El-Sayed et al., 2019). To date, 915 variants have been updated in the HGMD.

The complexity of pathogenesis and the overlap of phenotypes among subgroups within the same group, governed by numerous gene variants, present significant challenges for clinicians in early and accurate diagnosis for patients. Fortunately, the advantages of whole exome sequencing (WES) have provided a useful solution for identifying pathogenic variants, especially for multigenic diseases such as IEIs. In this study, we used WES to aid in the clinical diagnosis of patients suspected of having IEI. The results emphasize that genetic testing helps identify the abnormal gene and provides insight into the disease’s pathogenesis, enabling accurate determination of optimal treatment.

## Materials and methods

### Sample collection

Patients from six unrelated families were diagnosed with IEI at the Allergy, Immunology, and Rheumatology Department, Vietnam National Hospital of Pediatrics. The patients’ clinical and paraclinical characteristics were summarized in Tables 1, 2. Normal ranges for hematological, blood biochemical, and lymphocyte subtype parameters were referenced from Tosato et al. (2015), Esan (2016), Bayram et al. (2019). Peripheral blood samples were collected from patients for the WES analysis to identify the pathogenic variants in the patients.

**TABLE 1:** Clinical information of patients in the study.

Patient	Sex	Age of diagnosis	Respiratory inflammation	Pneumonia	Otitis media	Diarrhea	Sepsis	Dermatitis	Oral thrush	Meningitis	Skin abscess
IEI1	Male	16 days	​	x	​	​	x	​	x	​	​
IEI2	Male	6 years old	​	x	x	​	​	​	​	x	​
IEI3	Male	6 years old	X	x	x	​	​	​	​	x	​
IEI4	Female	5 years old	X	x	​	​	​	​	​	​	x
IEI5	Female	3 years old	​	x	x	x	​	​	​	​	x
IEI6	Male	4 months	X	​	x	x	x	x	x	​	​

x, presents the clinical symptoms that appear in the patient.

**TABLE 2:** Paraclinical characteristics in patients.

Patient	IEI1	IEI2	IEI3	IEI4	IEI5	IEI6
Blood formula indicators						
WBC (g/L) (normal range)	3.80	5.57 (4.50–13.50)	11.10 (4.50–13.50)	**12.40** (5.00–14.50)	**25.3** (5.00–14.50)	**3.18** (6.0–17.5)
Neut (g/L) (normal range)	1.91 (1.80–5.40)	2.34 (1.50–8.00)	7.20 (1.50–8.00)	**9.20** (1.50–8.00)	**17.6** (1.50–8.00)	**0.18** (1.5–8.5)
Lymp (g/L) (normal range)	**1.03** (2.90–9.10)	2.44 (1.50–6.80)	2.34 (1.50–6.80)	2.02 (1.50–7.00)	4.00 (1.50–7.00)	**1.09** (4.0–10.5)
Mono (g/L) (normal value)	0.87 (0.70)	0.56 (0.40)	1.52 (0.40)	0.68 (0.40)	1.64 (0.40)	1.75 (0.60)
EO (g/L) (normal value)	0.00 (0.2)	0.20 (0.20)	0.00 (0.20)	**0.41** (0.20)	**0.60** (0.20)	0.15 (0.30)
Plt (T/L)	198	408	328	207	657	854
Hb (g/dL)	100	97	120	130	69	95
Serum immunoglobulin concentrations						
IgA (g/L) (normal range)	**0.03** (0.067*–*0.246)	**0.000** (0.448*–*2.760)	**0.04** (0.448*–*2.760)	1.99 (0.357*–*1.920)	1.32 (0.357*–*1.920)	**0.02** (0.067*–*0.530)
IgM (g/L) (normal range)	**0.04** (0.152*–*0.685)	**0.001** (0.503*–*2.420)	**0.08** (0.503*–*2.420)	2.18 (0.587*–*1.980)	2.36 (0.587*–*1.980)	0.10 (0.269*–*1.300)
IgG (g/L) (normal range)	2.29 (2.170*–*9.810)	7.25 (4.830*–*15.800)	**2.17** (4.830*–*15.800)	12.20 (4.570*–*11.200)	12.40 (4.570*–*11.200)	**0.14** (2.170*–*9.810)
IgE (IU/mL) (normal value)	-(<60)	-(<15)	-(<15)	**3091.6** (<15)	**10,355** (<15)	-(<60)
Blood biochemical indicators						
Protein (g/L)	55.0	49.7	-	-	-	60.0
Albumin (g/L)	36.0	35.7	-	33.7	24.0	36.6
Urea (mmol/L)	1.7	2.7	-	2.6	2.7	4.5
Creatinine (μmol/L)	28.0	42.2	-	55.8	17.0	27.0
AST (U/L)	1,052	33.6	-	11.9	37.0	31.0
ALT (U/L)	234.0	43.4	-	71.8	7.8	68.0
CRP (mg/L)	1.2	1.1	-	247.0	20.2	48.0
Flow cytometry indicators						
CD3 (nx10^6^/L) (Normal range)	**34** (3180–5,401)	2,237 (1,239–2,611)	2,239 (1,239–2,611)	3278 (1,578–3707)	-	**514** (2,284–4,776)
CD4 (nx10^6^/L) (Normal range)	**24** (2,330–3617)	668 (646–1,515)	901 (646–1,515)	1,495 (870–2,144)	-	**249** (1,523–3472)
CD8 (nx10^6^/L) (Normal range)	**27.72** (712–1,361)	1,523 (365–945)	872 (365–945)	1,515 (472–1,107)	-	**226** (524–1,583)
CD19 (nx10^6^/L) (Normal range)	1,375 (315–1,383)	**0.87** (276–640)	**7.43** (276–640)	636 (434–1,274)	-	**15.8** (776–2,238)
CD56 (nx10^6^/L) (Normal range)	204 (201–870)	**107** (120–483)	**89** (120–483)	936 (155–565)	-	393 (230–801)

WBC: white blood cell; Neut: Neutrophil; Lymp: Lymphocyte; Mono: Monocyte; EO: eosinophils; Plt: Platele; Hb: Hemoglobin; AST: aspartate aminotransferase; ALT: alanine aminotransferase; CRP: *C-reactive protein;* Bold letters indicates values out of the normal range.

Reference ranges for serum immunoglobulin according to Tosato et al., 2015.

Reference ranges for hematological values according to Esan (2016).

Reference ranges for lymphocyte subsets according to Bayram et al., 2019.

### Ethics approval and consent to participate

The study was conducted in accordance with the Declaration of Helsinki and approved by the Ethics Committee of the Institute of Genome Research (Approval No. 03-2023/NCHG-HDDD). Written informed consent was obtained from the patient’s parents for the publication of any potentially identifiable images or data included in the article.

### WES and sanger sequencing

DNA was isolated from peripheral blood samples from patients and their families using a Qiagen DNA Blood Mini kit (QIAGEN, Hilden, Germany) according to the manufacturer’s guidelines. WES was performed on the Illumina sequencing machine (Illumina, CA, United States) using the SureSelect Human All Exon V7 kit with a library enriched using the SureselectXT Reagent kit (Agilent, Santa Clara, CA, United States). WES data with an average throughput depth of target regions of 150X, base quality thresholds with Q20 > 95% and Q30 > 90%, read alignment parameters, and variant caller filters with ReadPosRankSum < *−*8.0 were used for bioinformatic analysis. Data were aligned and compared with the human reference genome sequence version Hg19, thereafter, variants in genes associated with IEI were identified based on known and/or novel pathogenic variants, and those with minor allele frequency (MAF) < 0.001. Sanger sequencing was done on the ABI PRISM 3500 Genetic Analyzer machine (Thermo Fisher Scientific Inc., United States). The sequencing data were analyzed using BioEdit 7.2.5 software. Details of the analysis process were conducted as described in a previous study (Lien et al., 2024; Tran et al., 2024).

### Variant evaluation

The pathogenicity of novel variant was assessed using ACMG (The American College of Medical Genetics and Genomics) criteria and *in silico* predictive software such as EX-SKIP (https://ex-skip.img.cas.cz/), MaxEntScan (http://hollywood.mit.edu/burgelab/maxent/Xmaxentscan_scoreseq.html), NetGene2 (https://services.healthtech.dtu.dk/services/NetGene2-2.42/), and Spliceailookup (https://spliceailookup.broadinstitute.org/).

## Results

### Case presentation

Six patients from unrelated families were recruited for this study. None of the patients had a family history of IEIs; other family members, including parents and siblings, were healthy. Patient IEI1 was a 16-day-old boy admitted with pneumonia, sepsis, and oral thrush (Table 1). Clinical indicators (Table 2) showed that the levels of CD3^+^, CD4^+^, and CD8^+^ were very low at 34 × 10^6^/L (normal range is 3180–5,401 × 10^6^/L), 24 × 10^6^/L (normal range, 2,330–3617 × 10^6^/L), and 27.72 × 10^6^/L (normal range, 712–1,361 × 10^6^/L), respectively. The patient’s IgA and IgM levels were also low at 0.03 g/L (normal range, 0.067*–*0.246 g/L) and 0.04 g/L (normal range, 0.152*–*0.685 g/L), respectively, while IgG was within normal range at 2.29 g/L (normal range, 2.17*–*9.81 g/L). Patients IEI2 and IEI3 were two 6-year-old boys admitted with pneumonia, otitis media, meningitis, and recurrent respiratory infections. Both patients had low IgA level (0.000 g/L and 0.04 g/L, respectively, while normal range is 0.448–2.760 g/L) and low IgM level (0.001 g/L and 0.08 g/L, normal range is 0.503–2.420 g/L). In addition, a low IgG level 2.17 g/L (normal range, 4.830–15.800 g/L) was revealed in patient IEI3. Besides that, clinical indicators showed that the levels of CD19^+^ and CD56^+^ were very low in the patients. In IEI2 and IEI3 patients, CD19^+^ and CD56^+^ concentrations were 0.87 × 10^6^/L and 7.43 × 10^6^/L, respectively (normal range 434–1,274 × 10^6^/L), and 107 × 10^6^/L and 89 × 10^6^/L, respectively (normal range 155–565 × 10^6^/L). Patients IEI4 and IEI5 were two girls, aged 5 and 3 years old, with symptoms of respiratory infections, recurrent pneumonia, otitis media, diarrhea, and recurrent skin abscesses. Their IgE levels were high at 3091.6 IU/mL and 10,355 IU/mL, respectively (normal range <60 IU/mL). Patient IEI6 is a 4-month-old boy with respiratory tract infection, otitis media, diarrhea, sepsis, dermatitis, and oral thrush. In the initial laboratory result, blood counts of white blood cells, neutrophils, and lymphocytes were all very low at 3.18 × 10^9^/L, 0.18 × 10^9^/L, and 1.09 × 10^9^/L, respectively. In the patient, the levels of IgA (0.02 g/L with normal range, 0.067*–*0.530 g/L), IgG (0.14 g/L with normal range, 2.170–9.810 g/L), and CD3^+^, CD4^+^, CD8^+^, and CD19^+^ (514 × 10^6^/L normal range, 2,284–4,776 × 10^6^/L; 249 × 10^6^/L normal range, 1,523–3472 × 10^6^/L; 226 × 10^6^/L normal range, 524–1,583 × 10^6^/L; and 15.8 × 10^6^/L normal range, 776–2,238 × 10^6^/L, respectively) were all below normal thresholds. Patients were treated with antibiotics to reduce inflammation and IVIG therapy to boost immunity. Currently, no patients have been treated with HSCT.

### WES and variant evaluation

Six patients with clinical and paraclinical manifestations of IEI underwent WES to identify pathogenic variants in related genes. Screening results identified six variants, including: c.116-2A>G (in the *IL2RG* gene)*;* c.215delA, p.Asn72Ilefs*49 and c.83G>A, p.Arg28His (in the *BTK* gene); c.1110-3C>A (in the *STAT3* gene)*;* c.1114G>A, p.Glu372Lys (in the *STAT6* gene)*;* and c.526C>T, p.Arg176* (in the *NHEJ1* gene) (Table 3). Of these, the variants identified as pathogenic in the ClinVar database were c.116-2A>G (in the *IL2RG* gene)*;* c.215delA, p.Asn72Ilefs*49 and c.83G>A, p.Arg28His (in the *BTK* gene); c.1114G>A, p.Glu372Lys (in the *STAT6* gene)*;* and c.526C>T, p.Arg176* (in the *NHEJ1* gene); while c.1110-3C>A (in the *STAT3* gene) is novel variant in IEI patient. Variants were also assessed as pathogenic by ACMG assessment criteria with very strong evidence (PVS1 or PS), two or more moderate evidence (PM), and supporting evidence (PP). Two variants (c.1110-3C>A in *STAT3* and c.1114G>A in *STAT6*) were assessed as VUS by ACMG (with PM2 and PP3 supporting c.1110-3C>A in *STAT3,* and PM2, PP2, and PP3 supporting c.1114G>A in *STAT6*). However, the variant c.1114G>A in the *STAT6* gene has been reported to be pathogenic based on ClinVar, and a novel c.1110-3C>A variant in the *STAT3* genehas been assessed as the cause in the patient based on the prediction tools (Table 4, Supplementary Material). Assessing the pathogenicity of the c.1110-3C>A variant revealed that it led to “exon skipping” (with a score of −622.489 by EX-SKIP software) and was a damaging variant (according to MaxEntScan software with score 3.97 for mutant compaired 8.12 for wildtype) due to causing “acceptor loss” as determined by Fruitfy (with score 0.92 for mutant compaired 0.42 for wildtype), NetGene2 (with score 0.00 for mutant compaired 0.48 for wildtype), and Spliceailookup software (with score 0.00 for mutant compaired 0.73 for wildtype).

**TABLE 3:** Variants identified in patients in the study.

Patient	IEI1	IEI2	IEI3	IEI4	IEI5	IEI6
Gene	*IL2RG* OMIM#308380 Xq13.1	*BTK* OMIM#300300 Xq22.3	*BTK* OMIM#300300 Xq22.3	*STAT3* OMIM#102582 17q21.2	*STAT6* OMIM#601512 12q13.3	*NHEJ1* OMIM#611290 2q35
Hereditary	X-linked	X-linked	X-linked	AD	AD	AR
cDNA	c.116–2A>G	c.215delA	c.83G>A	c.1110–3C>A	c.1114G>A	c.526C>T
Protein	Splice acceptor	p.Asn72Ilefs*49	p.Arg28His	Splice acceptor	p.Glu372Lys	p.Arg176*
Zygosity	Hem	Hem	Hem	Het	Het	Hom
dbSNP	rs2147751146	rs886041148	rs128620185	Novel	rs2548028861	rs1304446470
ClinVar	RCV001377945.9 Likely pathogenic	RCV001784058.26 Pathogenic	RCV000012101.18 Pathogenic	-	RCV003333721 Pathogenic	RCV003494647 Pathogenic
ACMG	Pathogenic (PVS1, PP5, PM2)	Pathogenic (PVS1, PP5, PM2)	Pathogenic (PP5, PM1, PM5, PP3, PS3, PM2)	VUS (PP3, PM2)	VUS (PP3, PM2, PP2)	Pathogenic (PVS1, PP5, PM2)
Reference	-	Arunachalam et al. (2021)	Lee et al. (2010)	This study	Baris et al. (2023)	Dutrannoy et al., 2010 Ijspeert et al. (2016)

AD: autosomal dominant; AR: autosomal recessive; Hom: Homozygous; Hem: Hemizygous; Het: Heterozygous; VUS: variant of uncertain significance.

**TABLE 4:** Predicted results for the c.1110–3C>A variant in the *STAT3* gene.

*In silico* prediction tools	Wildtype	Mutant	Prediction
EX-SKIP	-	−622.489	Exon skipping
MaxEntScan	8.12	3.97	Damage variant
Fruitfy	0.92	0.42	Acceptor loss
NetGene2	0.48	-	Acceptor loss
Spliceailookup	-	0.73	Acceptor loss

## Discussion

In this study, based on clinical and genetic analysis, two patients (IEI1 and IEI6) were diagnosed with SCID, two patients (IEI2 and IEI3) with XLA, and two patients (IEI4 and IEI5) with HIES. Six variants, including: c.116-2A>G (in the *IL2RG* gene)*;* c.215delA, p.Asn72Ilefs*49 and c.83G>A, p.Arg28His (in the *BTK* gene); c.1110-3C>A (in the *STAT3* gene)*;* c.1114G>A, p.Glu372Lys (in the *STAT6* gene)*;* and c.526C>T, p.Arg176* (in the *NHEJ1* gene) were identified in the patients. The IEI1 patient had a very early age of onset at only 16 days old. Clinical findings indicated severe T-cell depletion and dysfunction with CD3^+^ at 34 × 10^6^/L (normal range is 3180–5,401 × 10^6^/L), CD4^+^ at 24 × 10^6^/L (normal range, 2,330–3617 × 10^6^/L), and CD8^+^ at 27.72 × 10^6^/L (normal range, 712–1,361 × 10^6^/L), and lymphocytes account for 1.03 × 10^9^/L (normal range, 2.9–9.1 × 10^9^/L). However, the B cell count CD19^+^ at 1,375 × 10^6^/L (normal range, 315–1,383 × 10^6^/L) and NK cell count CD56^+^ at 204 × 10^6^/L (normal range, 201–870 × 10^6^/L) were normal. The patient’s IgA and IgM levels were also low at 0.03 g/L (normal range, 0.067*–*0.246 g/L) and 0.04 g/L (normal range, 0.152*–*0.685 g/L), respectively, while IgG was within normal range (Table 2). The severe depletion and dysfunction of T cells is the cause of early disease onset and severe clinical symptoms in the patient. The patient experienced recurrent pneumonia, sepsis, and oral thrush and died at age one.

**FIGURE 1:**
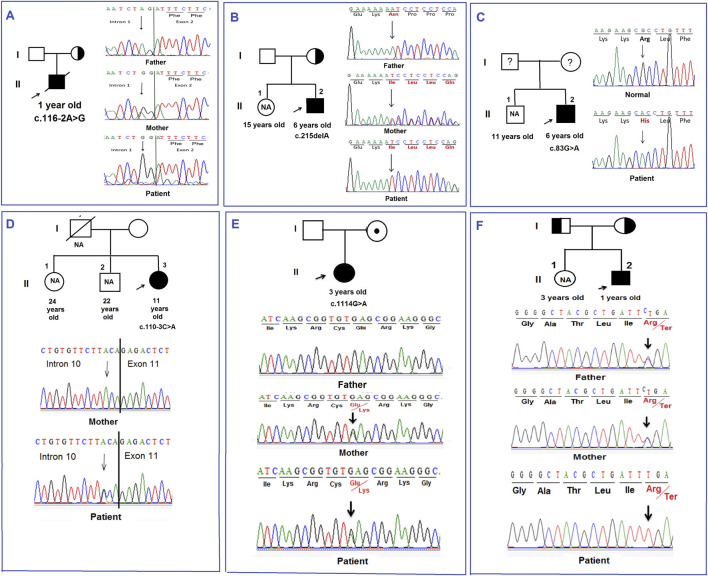
Results of Sanger sequencing analysis in the Vietnamese patients with IEI. **(A)** Family pedigree of the IEI1 patient and Sanger sequencing at the c.116-2A>G variant position in the *IL2RG* gene. **(B)** Family pedigree of the IEI2 patient and Sanger sequencing at the c.215delA, p.Asn72Ilefs*49 variant in the *BTK* gene. **(C)** Family pedigree of the IEI3 patient and Sanger sequencing at the c.83G>A, p.Arg28His variant in the *BTK* gene. **(D)** Family pedigree of the IEI4 patient and Sanger sequencing at the c.1110-3C>A variant in the *STAT3* gene. **(E)** Family pedigree of the IEI5 patient and Sanger sequencing at the c.1114G>A, p.Glu372Lys variant in the *STAT6* gene. **(F)** Family pedigree of the IEI6 patient and Sanger sequencing at the c.526C>T, p.Arg176* variant in the *NHEJ1* gene.

Genetic testing identified the patient as carrying the hemizygous c.116-2A>G (rs2147751146) variant in the *IL2RG* gene. Sanger sequencing revealed that the variant was inherited from his mother in a heterozygous state (Figure 1A). This is a pathogenic variant in the *IL2RG* gene, published in ClinVar under accession number RCV001377945.9, which causes X-SCID, a life-threatening IEI (Al Dossari and Kamal, 2015). The variant is also considered a pathogenic variant based on ACMG assessment criteria; however, there are no publications on this variant in IEI patients. Most infants with X-SCID exhibit severe, recurrent infections starting in the first few months of life, often accompanied by diarrhea and developmental delays, leading to death within the first year of life if their immune system is not restored through HSCT (Belaid et al., 2021). The IL-2R gamma chain acts as a common signaling kinase of cytokine receptor subtypes by cooperating with other JAK and STAT proteins, which are crucial in lymphocyte proliferation and differentiation. Therefore, pathogenic variants in the *IL2RG* gene resulted in a lack of T cells, natural killer (NK) cells, and inactive B lymphocytes (Hou et al., 2022). Genetic analysis results showed genotype-phenotype (T-B + SCID) concordance in our patient.

In the IEI6 patient, clinical symptoms, including respiratory tract infections, otitis media, diarrhea, sepsis, dermatitis, and oral thrush, appeared at the fourth month (Table 1). CD3^+^, CD4^+^, CD8^+^, and CD19^+^ levels were very low, with CD3^+^ at 514 × 10^6^/L (normal range, 2,284–4,776 × 10^6^/L); CD4^+^ at 249 × 10^6^/L (normal range, 1,523–3472 × 10^6^/L); CD8^+^ at 226 × 10^6^/L (normal range, 524–1,583 × 10^6^/L); and CD19^+^ at 15.8 × 10^6^/L (normal range, 776–2,238 × 10^6^/L). While CD56^+^ was within the normal range (CD56 ^+^ 393 × 10^6^/L, normal range, 230–801 × 10^6^/L). The patient’s white blood cell, neutrophil, and lymphocyte counts were also very low at 3.18 × 10^9^/L, 0.18 × 10^9^/L, and 1.09 × 10^9^/L, respectively, while the average values were WBC 6.0–17.5 × 10^9^/L; neutrophil 1.5–8.5 × 10^9^/L; lymphocyte 4.0–10.5 × 10^9^/L. The patient’s IgA and IgG levels were also low at 0.02 g/L (normal range, 0.067*–*0.530 g/L) and 0.14 g/L (normal range, 2.170–9.810 g/L), respectively, while IgM level was within normal range (Table 2). Low IgG level have also been reported in a SCID patient carrying a pathogenic variant of the *NHEJ1* gene, with disease onset at 6 months of age (Jamee et al., 2022). Genetic testing results revealed that the IEI6 patient carried the homozygous c.526C>T, p.Arg176* variant in the *NHEJ1* gene, which was inherited from both parents who carried the heterozygous variant (Figure 1F)*.* The genotype (*NHEJ1*) identified in the patient was consistent with the patient’s phenotype (T–B–SCID) in the IUIS classification (Poli et al., 2025). The c.526C>T, p.Arg176* variant in the *NHEJ1* gene was identified as pathogenic in ClinVar, with accession number RCV003494647, and has been reported in IEI patients (Dutrannoy et al., 2010; Ijspeert et al., 2016) (Table 3).

Patients IEI2 and IEI3 in our study both presented with clinical manifestations such as pneumonia, otitis media, and recurrent meningitis (Table 1). Both patients had low IgA and IgM levels: 0.000 g/L and 0.001 g/L (respectively, in the IEI2 patient) and 0.04 g/L and 0.08 g/L (respectively, in the IEI3 patient), whereas the normal IgA level was at 0.448–2.760 g/L and the normal IgM level was at 0.503–2.420 g/L (Table 2). In addition, the IEI3 patient had a low IgG level of 2.17 g/L (normal range, 4.830–15.800 g/L) and recurrent respiratory inflammation. Clinical indicators (Table 2) showed that the levels of CD19^+^ and CD56^+^ were low in the patients, with the CD19^+^ level at 0.87 × 10^6^/L and 7.43 × 10^6^/L (normal range is 434–1,274 × 10^6^/L), and the CD56^+^ level at 107 × 10^6^/L and 89 × 10^6^/L (normal range is 155–565 × 10^6^/L), respectively. XLA is a rare genetic disorder of B lymphocyte differentiation, characterized by an absence or low number of mature B cells, significantly reduced serum levels of immunoglobulin classes, and a lack of specific antibody production (Conley et al., 2009). Plebani et al. (2002) studied 73 Italian patients, and Winkelstein et al. (2006) studied 201 American patients, showing that recurrent otitis and pneumonia were prevalent symptoms in these patients. A study on 62 XLA patients reported by Lee et al. (2010) also showed that fifteen patients had severe infections, most of which were respiratory infections, including meningitis (n = 4), severe sepsis (n = 2), and pneumonia with effusion (n = 3).

**FIGURE 2:**
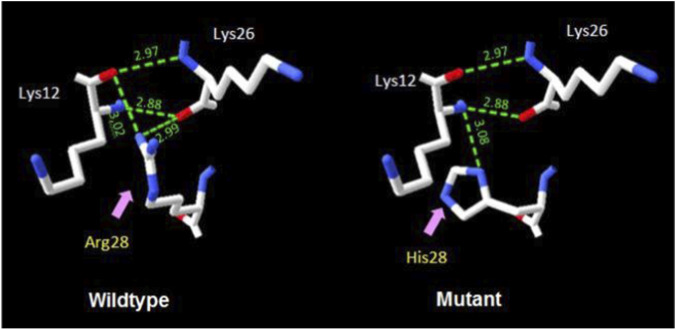
3-dimensional structure of the BTK protein at the position p.Arg28His, as predicted by Swiss-Pdb Viewer based on PDB: Q06187. The image shows that replacing the linear amino acid Arg (wildtype) with the cyclic amino acid His (mutant) resulted in the loss of a strong hydrogen bond between the amino acid His and the amino acid Lys at position 26.

Genetic testing revealed that patient IEI2 carried the known pathogenic variant c.215delA, p.Asn72Ilefs*49 (rs886041148, RCV001784058.26) and patient IEI3 carried the known pathogenic variant c.83G>A, p.Arg28His (rs128620185, RCV000012101.18) in the *BTK* gene (Table 3; Figures 1B,C). In addition, 3D structural analysis (Figure 2) (based on PDB: Q06187) revealed that the c.83G>A, p.Arg28His variant replaced the linear amino acid Arginine with the cyclic amino acid Histidine, thereby altering the hydrogen bonds between Histidine and Lysine at amino acid position 26. This alteration may have affected the protein’s tertiary structure and function, leading to the patient’s phenotype. These analytical results may provide further evidence regarding the influence of variants on disease expression in our patient.

Patient IEI4 was a 5-year-old girl, admitted to the hospital with symptoms of respiratory inflammation, pneumonia, recurrent skin abscess, and abnormally high IgE level 3091.6 IU/mL (normal range, <15 IU/mL) (Tables 1, 2). Eosinophils in the patient’s blood were also elevated, at 0.41 g/L compared to the normal level of 0.2 g/L. Patient IEI4 with an abnormally high serum IgE level is considered a characteristic manifestation of a diverse group of patients with IEI (Hashemi et al., 2017; Wu et al., 2018). Elevated serum IgE levels are important indicators for detecting underlying immune disorders, as IEIs are often misdiagnosed as common allergies (Galletta et al., 2024). Patients are susceptible to a variety of disorders, including recurrent dermatitis, eczema, skin abscesses, and pneumonia, depending on the clinical symptoms, which are primarily driven by different classifications of HIES (Gernez et al., 2018; Bergerson and Freeman, 2019; Tavakol et al., 2019; Minegishi, 2021). Eosinophilia (typically ≥0.7 g/L) was also observed in over 90% of patients in the study by Gernez et al. (2018). Furthermore, some immunodeficiency disorders can affect serum IgE levels, complicating diagnosis. Immunological features of STAT3-HIES have been reported to include normal serum IgG and IgM levels, extremely high serum IgE levels (>2000 IU/mL), serum eosinophilia, neutropenia, elevated γ-interferon and TNF-α levels, decreased memory T and B cells, and significantly reduced T cells (Benninghoff et al., 2008; Speckmann et al., 2008; Bergerson and Freeman, 2019). Therefore, genetic testing is necessary to determine the cause and select appropriate treatment (Al-Shaikhly and Ochs, 2019).

Genetic sequencing results in the IEI4 patient in our study revealed that the patient carried the c.1110-3C>A variant, a novel variant that not previously reported in the ClinVar database, in the *STAT3* gene (Figure 1D). This variant has been assessed as the cause in the patient based on the prediction tool (Table 4; Supplementary Material). STAT3, a DNA-binding protein located downstream of the epidermal growth factor receptor, plays a central role in signaling pathways induced by numerous cytokines, including IL-6, IL-10, IL-11, IL-17, IL-21, and IL-22. Therefore, STAT3 deficiency leads to increased production of several Th1 cytokines, such as IFNγ and TNFα, and decreased production of pro-inflammatory and anti-inflammatory cytokines regulated by IL-6 and IL-10 (O’Shea et al., 2013) resulting in immunosuppression in HIES. Additionally, STAT3, which is thought to play a role in regulating epithelial cell barrier function (Sonnenberg et al., 2011), is also disrupted in HIES. *STAT3* variants have been shown to lead to failure in the differentiation of CD4 Th17 cells (Milner et al., 2008), and defective Th17 responses play a central role in several immunodeficiency diseases (Puel et al., 2012). Previous studies shown that the structure of STAT3 comprises three main regions: the N-terminal region (1–355), the centromeric region (the DNA-binding domain) (355–555), and the C-terminal region (the SH2 region) (555–770). A large number of variants, mainly concentrated in the DNA-binding domain and the SH2 domain, have been identified as causing negative dominance effects in STAT3 (Renner et al., 2008). The c.1110-3C>A variant in the *STAT3* gene in the IEI4 patient is located in the centromeric region and may affect the SH2 region due to “exon skipping”, which could be the cause of the disease in this patient.

Patient IEI5 was a 3-year-old girl, admitted to the hospital with symptoms of pneumonia, otitis media, skin abscess, and recurrent diarrhea (Table 1). The results of the paraclinical tests showed that white blood cell, neutrophil, and eosinophil counts were higher at 25.3 g/L, 17.6 g/L, and 0.6 g/L in the patient compared to the normal level of 5.0–14.5 g/L*,* 1.5–8.0 g/L, and 0.2 g/L, respectively (Table 2). In the immunologic workup, extremely high serum high IgE level 10,355 IU/mL (normal range, <15 IU/mL) was observed in the patient. The genetic study in the patient showed a heterozygous variant, c.1114G>A (p.Glu372Lys), in the *STAT6* gene. This variant has been reported previously as the pathogenic variant in the ClinVar database under accession number RCV003333721. Previous studies have shown that STAT6 is widely expressed in many cell types (such as T cells and B cells) (Goenka and Kaplan, 2011) and is closely associated with the development, differentiation, class switching, metabolism, and morphology of B cells. The role of STAT6 in IL-4 activity is crucial. STAT6 deficiency leads to IL-4 deficiency, disrupting Th2 differentiation and Ig class switching (Wang et al., 2021). Baris et al. (2023) reported a case of a child with a missense mutation in the DNA-binding domain of STAT6 (c.1114G>A, p.Glu372Lys) who presented similar symptoms with severe atopic dermatitis, eosinophilia, and elevated IgE. In the patient, symptoms were present from infancy, such as severe atopic eczema, incessant itching, severe growth retardation, systemic lymphadenopathy, and pneumonia. To date, there have been four reports, describing 13 families with 21 affected patients, and have demonstrated that STAT6-GOF was a new and distinct monogenic primary allergic disorder (Baris et al., 2023; Sharma et al., 2023; Suratannon et al., 2023; Takeuchi et al., 2023).

## Conclusion

In summary, we analyzed the genotypes and phenotypes of six Vietnamese IEI patients, including two patients with SCID, two with XLA, and two with HIES. In this study, WES was used to identify potentially pathogenic variants in the patients. The analysis revealed six variants in five different genes, including c.116-2A>G (in the *IL2RG* gene); c.215delA, p.Asn72Ilefs*49 and c.83G>A, p.Arg28His (in the *BTK* gene); c.1110-3C>A (in the *STAT3* gene); c.1114G>A, p.Glu372Lys (in the *STAT6* gene); and c.526C>T, p.Arg176* (in the *NHEJ1* gene). Of these, the variant c.1110-3C>A in the *STAT3* gene was newly identified in an IEI patient. Genotype and phenotypic analysis results showed that pathogenic variants in different genes played a decisive role in the severity of symptom manifestation in the patients. Comparison of clinical and paraclinical outcomes demonstrated that relying solely on patient phenotype may not fully reflect the patient’s genotype. These results highlight the complex pathogenesis of IEIs and demonstrate that WES is a useful tool for detecting pathogenic variants in polygenic diseases like IEIs. The results of this study emphasize the importance of accurate diagnosis that improves treatment outcomes and enhances the quality of care for individuals with IEIs.

## Data Availability

The original contributions presented in the study are included in the article/Supplementary Material, further inquiries can be directed to the corresponding author.
