# Supplementary material for: Case Report: A case series of using whole exome sequencing to detect novel variants in Vietnamese patients with inborn errors of immunity
Source: Front Genet. 2026 May 28;17:1818952. doi: 10.3389/fgene.2026.1818952 (PMC13252911; doi:10.3389/fgene.2026.1818952)

**Prediction results using *in silico* tools for splicing variant c.1110-3C>A in the *STAT3* gene**

**1. Prediction result using the EX-SKIP tool**


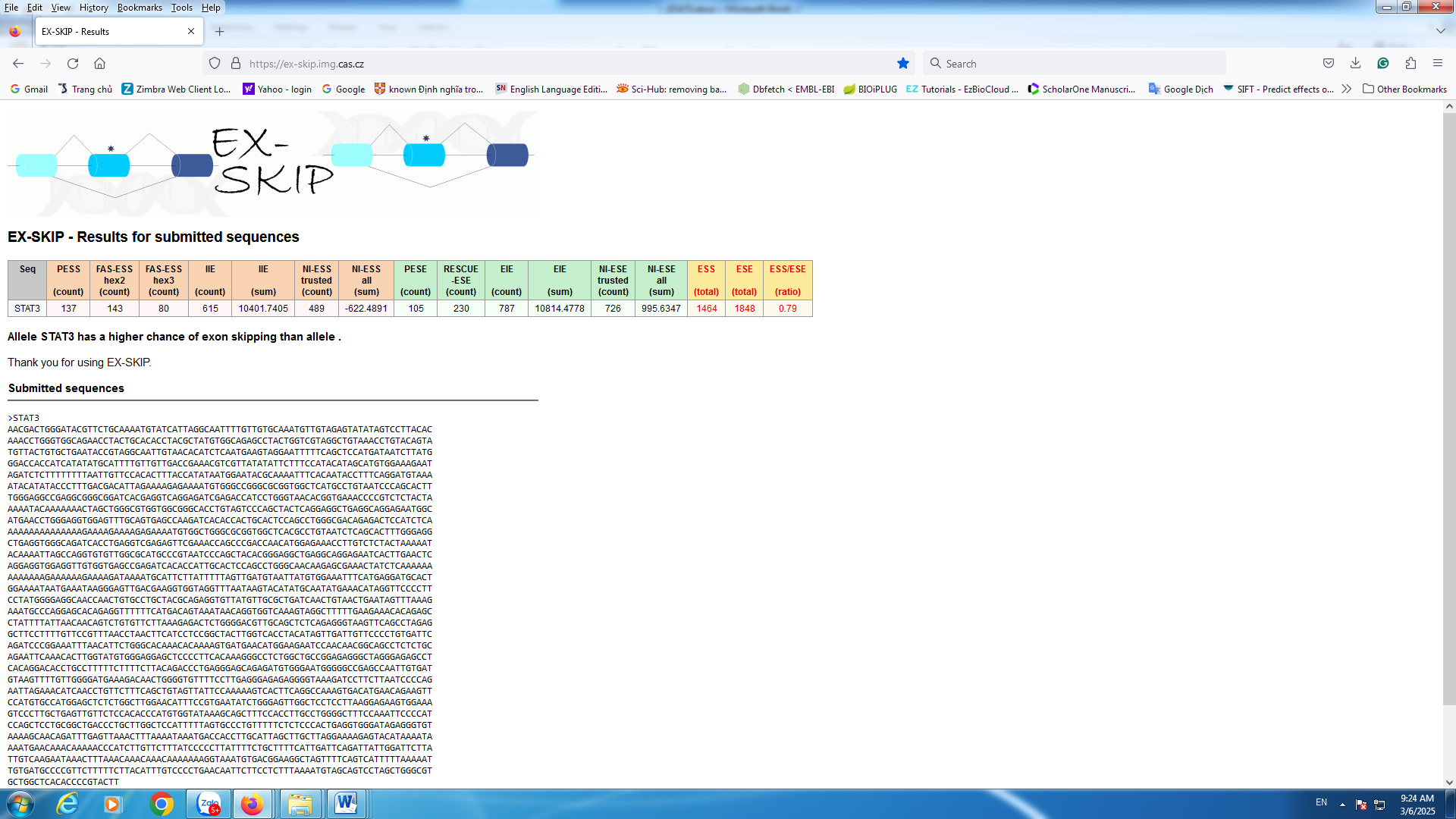


**2. Prediction result using the Fruitfy tool**

**Donor site predictions for wild :**

**Start End Score Exon Intron**
 502 516 0.85 tcacgaggtcaggag
 526 540 0.58 atcctgggtaacacg
 888 902 0.76 tagccaggtgtgttg
 971 985 0.55 ggttgtggtgagccg
 1082 1096 0.62 agttgatgtaattat
 1148 1162 0.63 gaaggtggtaggttt
 1417 1431 1.00 tcagagggtaagttc
 1610 1624 0.82 acacttggtatgtgg
 1754 1768 0.97 ttgtgatgtaagttt
 2138 2152 0.56 cactgaggtgggata
 2352 2366 0.99 aaaaaaggtaaatgt

**Acceptor site predictions for wild :**

**Start End Score Intron Exon**
 286 326 0.53 atatattctttccatacatagcatgtggaaagaatagatct
 372 412 0.53 aaatttcacaatacctttcaggatgtaaaatacatataccc
 1063 1103 0.54 aaaatgcattcttatttttagttgatgtaattatgtggaaa
 **1373 1413 0.92** caacagtctgtgttcttacagagactctggggacgttgcag
 1502 1542 0.40 gattgttcccctgtgattcagatcccggaaatttaacattc
 1692 1732 0.99 tgcctttttcttttcttacagaccctgagggagcagagatg
 1820 1860 0.74 aagatccttcttaatccccagaattagaaacatcaacctgt
 1848 1888 0.57 aacatcaacctgttctttcagctgtagttattccaaaaagt
 2099 2139 0.54 cctgcttggctccatttttagtgccctgtttttctctccca
 2285 2325 0.78 ttctgcttttcattgattcagattattggattcttattgtc

**Donor site predictions for mutant :**

**Start End Score Exon Intron**
 502 516 0.85 tcacgaggtcaggag
 526 540 0.58 atcctgggtaacacg
 888 902 0.76 tagccaggtgtgttg
 971 985 0.55 ggttgtggtgagccg
 1082 1096 0.62 agttgatgtaattat
 1148 1162 0.63 gaaggtggtaggttt
 1417 1431 1.00 tcagagggtaagttc
 1610 1624 0.82 acacttggtatgtgg
 1754 1768 0.97 ttgtgatgtaagttt
 2138 2152 0.56 cactgaggtgggata
 2352 2366 0.99 aaaaaaggtaaatgt

**Acceptor site predictions for mutant :**

**Start End Score Intron Exon**
 286 326 0.53 atatattctttccatacatagcatgtggaaagaatagatct
 372 412 0.53 aaatttcacaatacctttcaggatgtaaaatacatataccc
 1063 1103 0.54 aaaatgcattcttatttttagttgatgtaattatgtggaaa
 **1373 1413 0.47** caacagtctgtgttcttaaagagactctggggacgttgcag
 1502 1542 0.40 gattgttcccctgtgattcagatcccggaaatttaacattc
 1692 1732 0.99 tgcctttttcttttcttacagaccctgagggagcagagatg
 1820 1860 0.74 aagatccttcttaatccccagaattagaaacatcaacctgt
 1848 1888 0.57 aacatcaacctgttctttcagctgtagttattccaaaaagt
 2099 2139 0.54 cctgcttggctccatttttagtgccctgtttttctctccca
 2285 2325 0.78 ttctgcttttcattgattcagattattggattcttattgtc

**3. Prediction result using the MaxEntscan tool**

>wild type

AACAGTCTGTGTTCTTA**C**AGAGA MAXENT: 8.12 MM: 6.94 WMM: 7.03

>mutant

AACAGTCTGTGTTCTTA**A**AGAGA MAXENT: 3.97 MM: 2.73 WMM: 3.66

**4. Prediction result using the NetGene2 v. 2.4 tool**

**The sequence: wild has the following composition:**

Length: 2501 nucleotides.

29.3% A, 21.0% C, 23.4% G, 26.3% T, 0.0% X, 44.4% G+C

Donor splice sites, direct strand

---------------------------------

pos 5'->3' phase strand confidence 5' exon intron 3'

895 0 + 0.37 AATTAGCCAG^GTGTGTTGGC

1155 0 + 0.49 GACGAAGGTG^GTAGGTTTAA

1424 2 + 0.62 CTCTCAGAGG^GTAAGTTCAG

1617 0 + 0.71 CAAACACTTG^GTATGTGGGA

1761 0 + 0.39 CAATTGTGAT^GTAAGTTTTG

2359 0 + 0.63 ACAAAAAAAG^GTAAATGTGA

Donor splice sites, complement strand

-------------------------------------

pos 3'->5' pos 5'->3' phase strand confidence 5' exon intron 3'

1495 1007 0 - 0.55 AATCAACTAT^GTAGGTGACC

385 2117 1 - 0.32 ATCCTGAAAG^GTATTGTGAA

84 2418 2 - 0.00 TGCCACCCAG^GTTTGTGTAA

Acceptor splice sites, direct strand

------------------------------------

pos 5'->3' phase strand confidence 5' intron exon 3'

95 0 + 0.07 TGGGTGGCAG^AACCTACTGC

**1393 1 + 0.48 GTTCTTACAG^AGACTCTGGG**

1712 0 + 0.77 TTTCTTACAG^ACCCTGAGGG

2144 2 + 0.25 TCCCACTGAG^GTGGGATAGA

Acceptor splice sites, complement strand

----------------------------------------

pos 3'->5' pos 5'->3' phase strand confidence 5' intron exon 3'

2335 167 0 - 0.14 TTGTTTAAAG^TTTATTCTTG

2140 362 2 - 0.56 CCCACCTCAG^TGGGAGAGAA

1983 519 0 - 0.25 TCTCCTTAAG^GAGGAGCCAA

1801 701 2 - 0.15 CTCCCTCAAG^GAAAACACCC

1716 786 2 - 0.33 GCTCCCTCAG^GGTCTGTAAG

1542 960 2 - 0.44 TTGTGCCCAG^AATGTTAAAT

1119 1383 0 - 0.26 TATTTTCCAG^TGCATCCTCA

717 1785 0 - 0.16 TTTTTTTGAG^ATGGAGTCTC

293 2209 1 - 0.18 GTATGGAAAG^AATATATAAC

**The sequence: mutant has the following composition:**

Length: 2501 nucleotides.

29.3% A, 21.0% C, 23.4% G, 26.3% T, 0.0% X, 44.3% G+C

Donor splice sites, direct strand

---------------------------------

pos 5'->3' phase strand confidence 5' exon intron 3'

895 0 + 0.37 AATTAGCCAG^GTGTGTTGGC

1155 0 + 0.49 GACGAAGGTG^GTAGGTTTAA

1424 1 + 0.54 CTCTCAGAGG^GTAAGTTCAG

1617 0 + 0.71 CAAACACTTG^GTATGTGGGA

1761 0 + 0.39 CAATTGTGAT^GTAAGTTTTG

2359 0 + 0.63 ACAAAAAAAG^GTAAATGTGA

Donor splice sites, complement strand

-------------------------------------

pos 3'->5' pos 5'->3' phase strand confidence 5' exon intron 3'

1495 1007 0 - 0.55 AATCAACTAT^GTAGGTGACC

385 2117 1 - 0.32 ATCCTGAAAG^GTATTGTGAA

84 2418 2 - 0.00 TGCCACCCAG^GTTTGTGTAA

Acceptor splice sites, direct strand

------------------------------------

pos 5'->3' phase strand confidence 5' intron exon 3'

95 0 + 0.07 TGGGTGGCAG^AACCTACTGC

1712 0 + 0.77 TTTCTTACAG^ACCCTGAGGG

2144 2 + 0.25 TCCCACTGAG^GTGGGATAGA

Acceptor splice sites, complement strand

----------------------------------------

pos 3'->5' pos 5'->3' phase strand confidence 5' intron exon 3'

2335 167 0 - 0.14 TTGTTTAAAG^TTTATTCTTG

2140 362 2 - 0.56 CCCACCTCAG^TGGGAGAGAA

1983 519 0 - 0.25 TCTCCTTAAG^GAGGAGCCAA

1801 701 2 - 0.15 CTCCCTCAAG^GAAAACACCC

1716 786 2 - 0.33 GCTCCCTCAG^GGTCTGTAAG

1542 960 2 - 0.43 TTGTGCCCAG^AATGTTAAAT

1119 1383 0 - 0.26 TATTTTCCAG^TGCATCCTCA

717 1785 0 - 0.16 TTTTTTTGAG^ATGGAGTCTC

293 2209 1 - 0.18 GTATGGAAAG^AATATATAAC

**5. Prediction result using the SpliceAI tool**


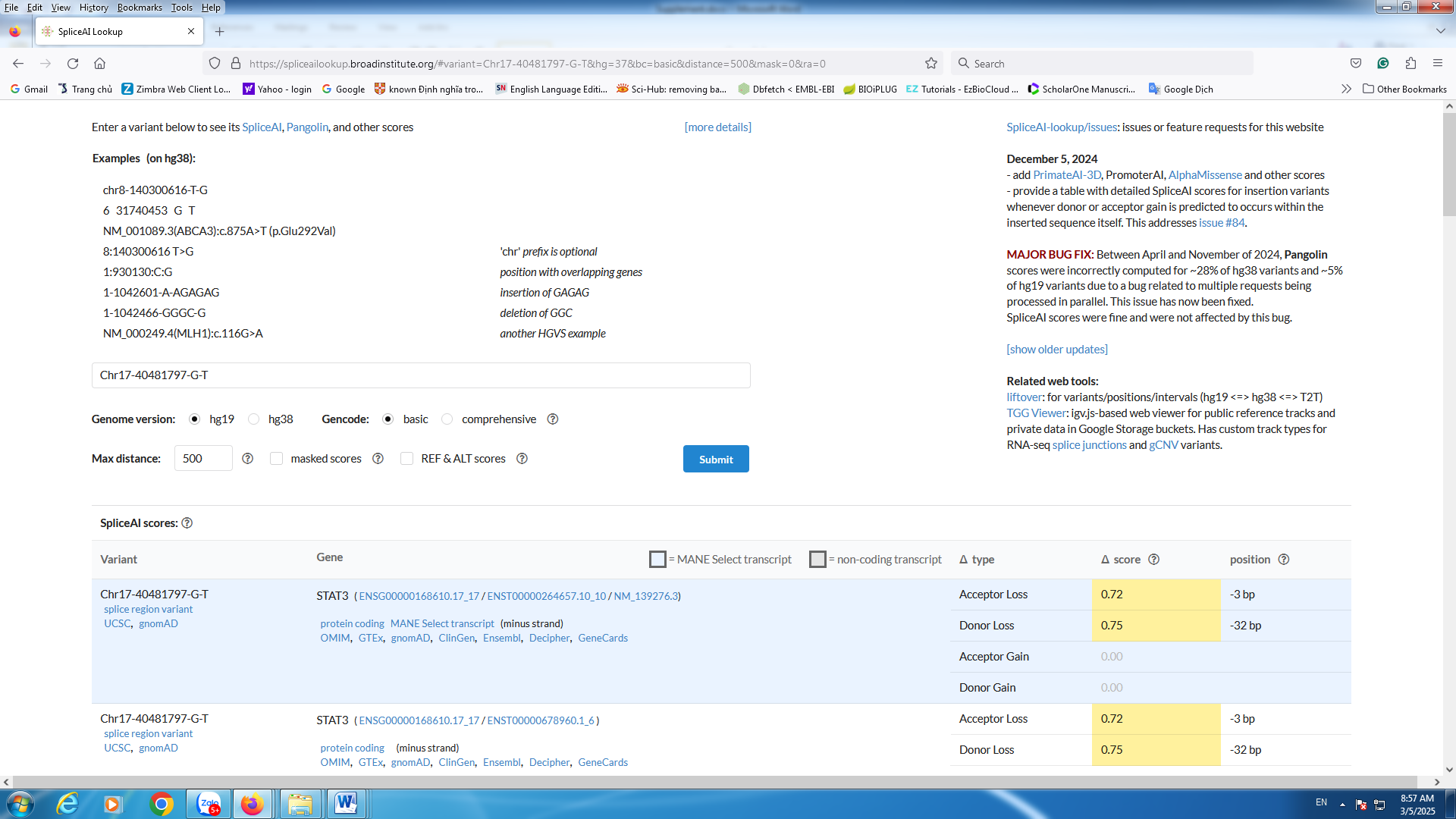


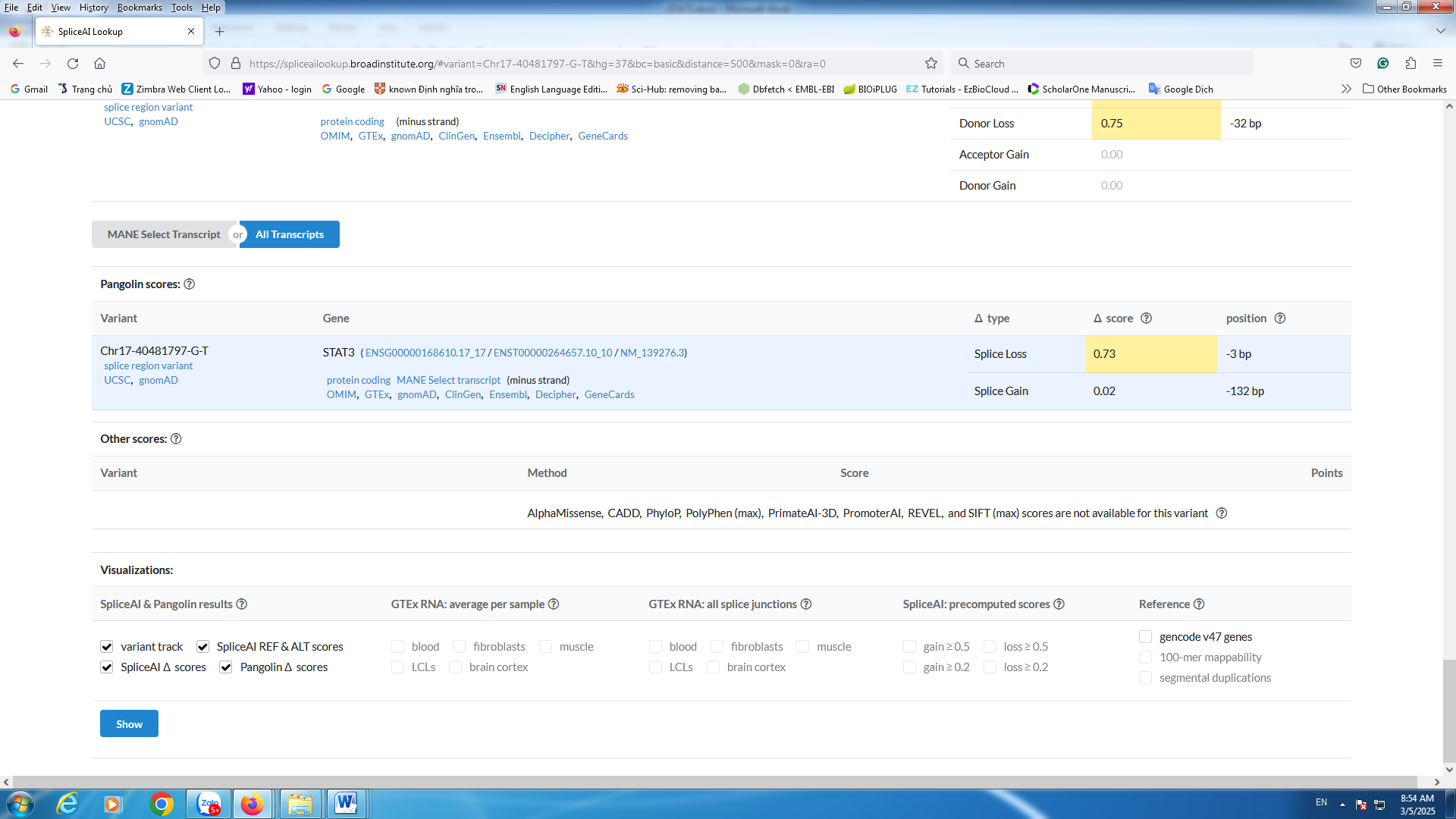

Supplement: Supplementary file 1 [file DataSheet1.docx]
